# Prophylactic treatment of rapamycin ameliorates naturally developing and episode -induced heterotopic ossification in mice expressing human mutant ACVR1

**DOI:** 10.1186/s13023-020-01406-8

**Published:** 2020-05-24

**Authors:** Hirotsugu Maekawa, Shunsuke Kawai, Megumi Nishio, Sanae Nagata, Yonghui Jin, Hiroyuki Yoshitomi, Shuichi Matsuda, Junya Toguchida

**Affiliations:** 1 Department of Cell Growth and Differentiation, Center for iPS Cell Research and Application, Kyoto University, Kyoto, Japan; 2 Department of Orthopaedic Surgery, Graduate School of Medicine, Kyoto University, Kyoto, Japan; 3 Department of Regeneration Sciences and Engineering, Institute for Frontier Life and Medical Sciences, Kyoto University, Kyoto, Japan; 4 Institute for Advancement of Clinical and Translational Sciences, Kyoto University Hospital, Kyoto University, Kyoto, Japan

**Keywords:** Fibrodysplasia ossificans progressiva, Heterotopic ossification, Rapamycin

## Abstract

**Background:**

Fibrodysplasia ossificans progressiva (FOP) is a rare autosomal-dominant disease characterized by heterotopic ossification (HO) in soft tissues and caused by a mutation of the *ACVR1A/ALK2* gene. Activin-A is a key molecule for initiating the process of HO via the activation of mTOR, while rapamycin, an mTOR inhibitor, effectively inhibits the Activin-A-induced HO. However, few reports have verified the effect of rapamycin on FOP in clinical perspectives.

**Methods:**

We investigated the effect of rapamycin for different clinical situations by using mice conditionally expressing human mutant ACVR1A/ALK2 gene. We also compared the effect of rapamycin between early and episode-initiated treatments for each situation.

**Results:**

Continuous, episode-independent administration of rapamycin reduced the incidence and severity of HO in the natural course of FOP mice. Pinch-injury induced HO not only at the injured sites, but also in the contralateral limbs and provoked a prolonged production of Activin-A in inflammatory cells. Although both early and injury-initiated treatment of rapamycin suppressed HO in the injured sites, the former was more effective at preventing HO in the contralateral limbs. Rapamycin was also effective at reducing the volume of recurrent HO after the surgical resection of injury-induced HO, for which the early treatment was more effective.

**Conclusion:**

Our study suggested that prophylactic treatment will be a choice of method for the clinical application of rapamycin for FOP.

## Introduction

Fibrodysplasia ossificans progressiva (FOP) (OMIM#135100) is a rare autosomal dominant hereditary disease characterized by congenital malformations of the great toes and progressive heterotopic ossification (HO) in fibrous tissues such as skeletal muscles, tendons, and ligaments. The prevalence has been estimated at approximately one in 2 million people [1], but a recent report using the nation-wide surveillance system in France revealed a much higher rate [2]. Although the clinical course of each patient varies considerably, the typical course of new HO is initiated by the appearance of localized painful swelling (flare-up) followed by the formation of bone tissue in the swollen site several weeks or months later [2–4]. Corticosteroids have been used to reduce the inflammation at the flare-up, but their effect at inhibiting the HO is limited [2, 5].

The disease-causing gene of FOP is *ACVR1A/ALK2* (activin receptor 1A/activin-like kinase-2), which encodes a type I receptor for bone morphogenetic protein (BMP) [6]. More than 95% of patients carry an identical missense mutation (c.617G > A; p.R206H) [7]. Histological examinations have demonstrated that HO is initiated by the infiltration of inflammatory cells, followed by the proliferation of myofibroblastic cells, the formation of neovascularity, the transformation of myofibroblastic tissues into chondroid tissues, and finally endochondral bone formation [3]. Although each step is important, we hypothesize that the most critical step is the transformation of myofibroblastic tissues to chondroid tissues, which is not observed in normal inflammatory processes after injury. To identify the factors responsible for this transformation, we established induced pluripotent stem cells (iPSCs) from FOP-patients (FOP-iPSCs) [8] and rescued FOP-iPSCs (resFOP-iPSCs), in which the mutant sequence was replaced with the normal sequence [9]. We induced these iPSCs to mesenchymal stromal cells (iMSCs) and discovered that Activin-A, which induces TGFβ signaling in normal cells, induces BMP signaling via FOP-ACVR1A to promote the chondrogenic differentiation of iMSCs derived from FOP patients (FOP-iMSCs) and to initiate HO by FOP-iMSCs in vivo [10]. Around the same time, another group identified Activin-A as a key molecule for HO in FOP using FOP-ACVR1 conditional knock-in mice [11].

Activin-A is a member of the TGFβ protein family and transduces TGFβ signaling by binding to ACVR1B/ALK4 or ACVR1C/ALK7 [12]. However, in the case of FOP patient cells, Activin-A induces both TGFβ and BMP signaling to initiate the biological process of HO. Using a reporter construct of the aggrecan gene as a surrogate marker for chondrogenic differentiation, we identified rapamycin, an mTOR inhibitor, as the most effective drug for inhibiting the Activin-A-induced chondrogenesis of FOP-iMSCs [13]. We further found that rapamycin suppressed Activin-A-triggered HO in FOP-ACVR1 conditional transgenic mice and inhibited the HO formed by FOP-iMSCs in vivo [13]. Rapamycin has been used as an immunosuppressant after organ transplantation for thousands of patients and also as an anti-proliferative drug for kaposiform hemangioendothelioma and lymphangioleiomyomatosis [14, 15]. Therefore, it is a rational candidate for a clinical trial for FOP.

Although FOP is progressive, the clinical course is not a simple time-dependent course, but rather a step-wise progression [2–4]. It has been well documented that HO occurs after the episode of localized painful swelling (flare-up), which is initiated by traumatic injury or spontaneously [2–4]. Recent historical analysis, however, revealed that approximately 40% of HO in FOP patients forms without flare-up, and patients failed to describe when the disease started to exaggerate [16]. This unrecognized progression should be seriously considered when designing a clinical trial of possible therapeutic drugs for FOP. Another important issue to be addressed is whether rapamycin can suppress recurrent HO after surgical resection, which is the only way to restore the joint function but also risks recurrence at the surgical site and exaggeration of the disease systemically [2, 4].

To design the appropriate schedule for administration, here we investigated the effect of rapamycin for FOP by mimicking non-flare-up, flare-up-induced, and surgery-induced HO in mice that conditionally express human mutant *ACVR1A/ALK2* gene.

## Methods

### FOP-ACVR1 conditional transgenic mice

The establishment of FOP-ACVR1 conditional transgenic mice (hereafter called FOP-ACVR1 mice) was reported previously [13], and 13- to 17-week-old female mice were used in the experiments in this study. To induce the expression of human mutant ACVR1A/ALK2, mice were fed water supplemented with 2 mg/ml doxycycline (DOX) and 10 mg/ml sucrose. The age and body weight at the start point of each experiment were matched between groups. All animal experiments were approved by the institutional animal committee of Kyoto University.

### Treatment with rapamycin

Rapamycin was purchased from MidChem Express and dissolved in 10% DMSO with 0.5% w/v methylcellulose 400. Rapamycin was injected intraperitoneally at 5 mg/kg once a day, 5 times a week. Mice in the vehicle-treated group received the same solution without rapamycin. All mouse experiments and analyses of the results were performed under blinded condition.

### Pinch-injury

Under isoflurane anesthesia (5% for induction, 2–3% for maintenance) (Abbvie Limited, Berkshire, UK), a skin incision (approximately 1.5 cm) was made on the lateral surface of the left hindlimb, and the left gastrocnemius muscle was exposed. The middle portion of the muscle was pinched by a tissue forceps for 5 sec, and then the skin was closed.

### Surgical resection of pinch-injury-induced HO

Twenty-one days after the pinch-injury, the injured sites were exposed under anesthesia, and ossified tissues were resected with a high-speed drill and bipolar electrocautery under loupe magnification as much as possible.

### X-ray and micro–computed tomography (μCT) analyses

For X-ray and μCT imaging, mice were anesthetized with a mixture of medetomidine, midazolam, and butorphanol. X-ray images were scanned using DX-50 (Faxitron Bioptics, Tuscon, AZ, USA). μCT images were scanned using an X-ray CT system (inspeXio SMX-100CT, Shimadzu, Kyoto) and analyzed with TRI/3D-BON software (Ratoc System Engineering Co., Ltd., Tokyo, Japan) according to the manufacturer’s instructions. Investigation was completed under a blinded condition of the experimental groups.

### Histochemical analyses

Collected tissue samples were fixed with 4% paraformaldehyde for 48 h, embedded in paraffin, sectioned, and stained with hematoxylin and eosin (H&E), Safranin O, and von Kossa.

### Quantitative RT-PCR analyses

After euthanizing the mice by CO_2_, the spleens were extirpated and homogenized using a Multi-Beads Shocker (Yasui Kikai Corporation, Osaka, Japan) according to the manufacturer’s instructions. Total RNA was extracted from homogenized tissues using Sepasol-RNA I Super G (Nacalai tesque, Kyoto, Japan) and the RNeasy Kit (QIAGEN, Valencia, CA, USA) and treated with the DNase-One Kit (QIAGEN) to remove genomic DNA. cDNA was synthesized using ReverTra Ace qPCR RT Master Mix (TOYOBO CO., LTD., Osaka, Japan). Quantitative PCR (qPCR) was performed with Thunderbird SYBR qPCR Mix (TOYOBO) and analyzed with the QuantiStudio 12 K Flex Real-time PCR System (Applied Biosystems, Foster city, CA, USA). The primer sequences are described in Additional file 1: Table S1. All data (relative expression) were normalized to *Gapdh*.

### Statistical analysis

Statistical analysis was performed using JMP Pro® 14 (SAS Institute Inc., Cary, NC, USA). The statistical significance was calculated using the Student’s t-test, Dunnett test, or Wilcoxon sum rank test, as described in the corresponding figure legends. Survival was assessed by log-rank. Statistical significance is indicated as *p < 0.05 or **p < 0.01.

## Results

### Rapamycin inhibits HO in the natural course of FOP-ACVR1 mice

**Fig. 1:**
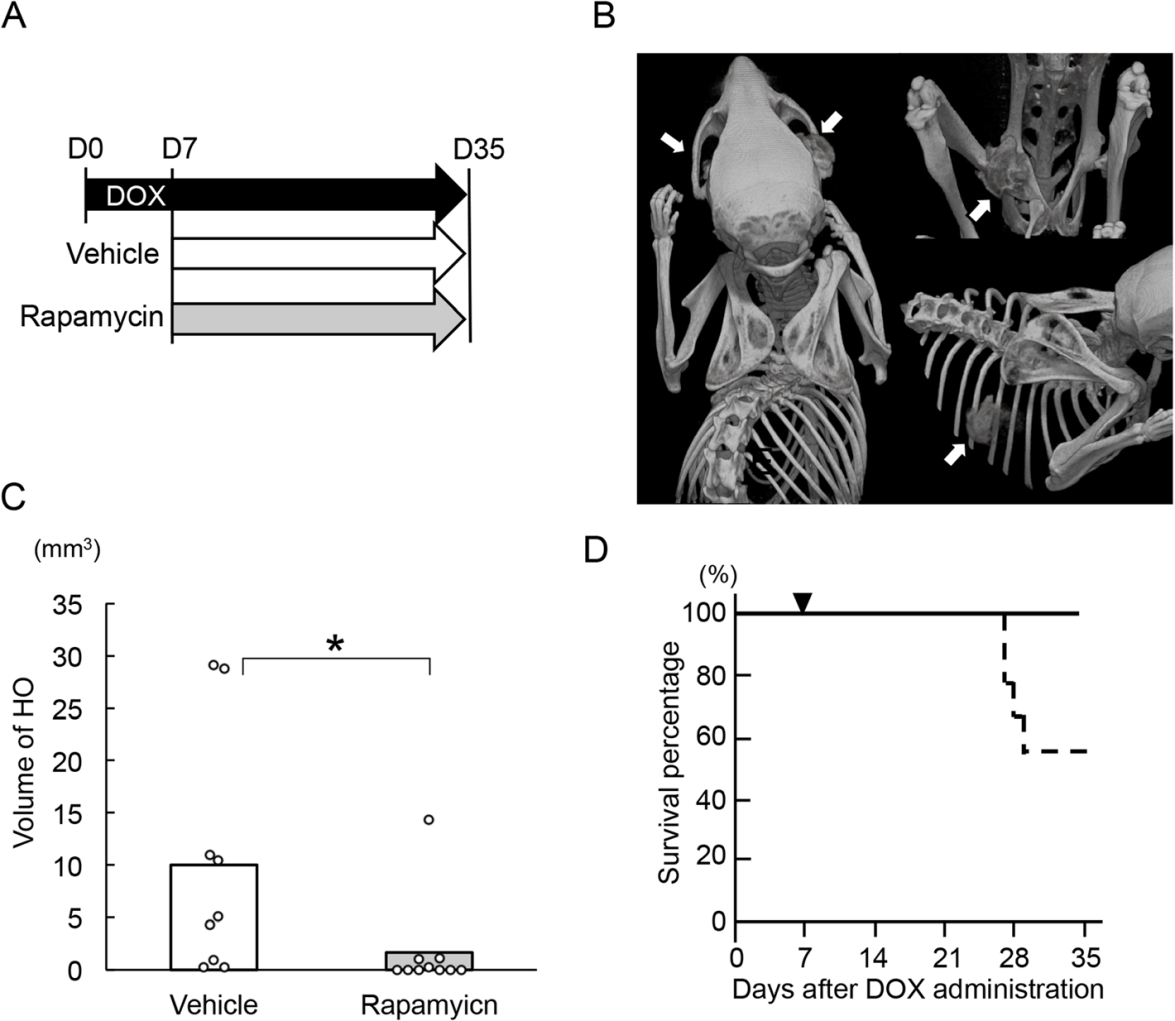
Rapamycin inhibits HO in the natural course of FOP-ACVR1 mice. **a**. Schematic view of the experiments. Each treatment was initiated 7 days after DOX administration. **b**. Representative μCT images of vehicle-treated mice. Arrows indicate HO. **c**. Quantification of HO volume on Day 35. Vehicle group: *n* = 9, rapamycin group: *n* = 10; *, *p* < 0.05, Wilcoxon rank sum test. **d**. Kaplan-Meier survival curves of vehicle-treated (dotted line) and rapamycin-treated (solid line) FOP-ACVR1 mice. ▼ indicates the first day of treatment. Vehicle group: *n* = 9, rapamycin group: *n* = 10; *p* < 0.05, log rank test

Singly-housed FOP-ACVR1 mice were fed DOX for 7 days and then randomly assigned to either the vehicle-treated (*n* = 9) or rapamycin-treated group (*n* = 10) (Fig. 1a, Table 1). The formation of HO was analyzed by whole-body CT on day 35 after DOX treatment or when the mice died. HO at the ribs, jaw, and hips was observed in nearly half of the vehicle-treated mice (Fig. 1b), and at least one site in all mice (Table 1). In the rapamycin-treated group, the incidence of HO was reduced (Table 1), and the average volume of HO was significantly smaller than that of the vehicle-treated group (Fig. 1c). Total bone volume was not significantly different between the two groups (Additional file 2: Fig. S1A). The average body weight of vehicle-treated mice tended to be lower at the late stage, but was not significantly different from that of rapamycin-treated mice (Additional file 2: Fig. S1B). All rapamycin-treated mice were alive by day 35, whereas four out of nine vehicle-treated mice died during this period (Fig. 1d), probably due to locking of the jaw, which was frequently observed in those mice (Fig. 1b, Table 1).

**Table 1:**
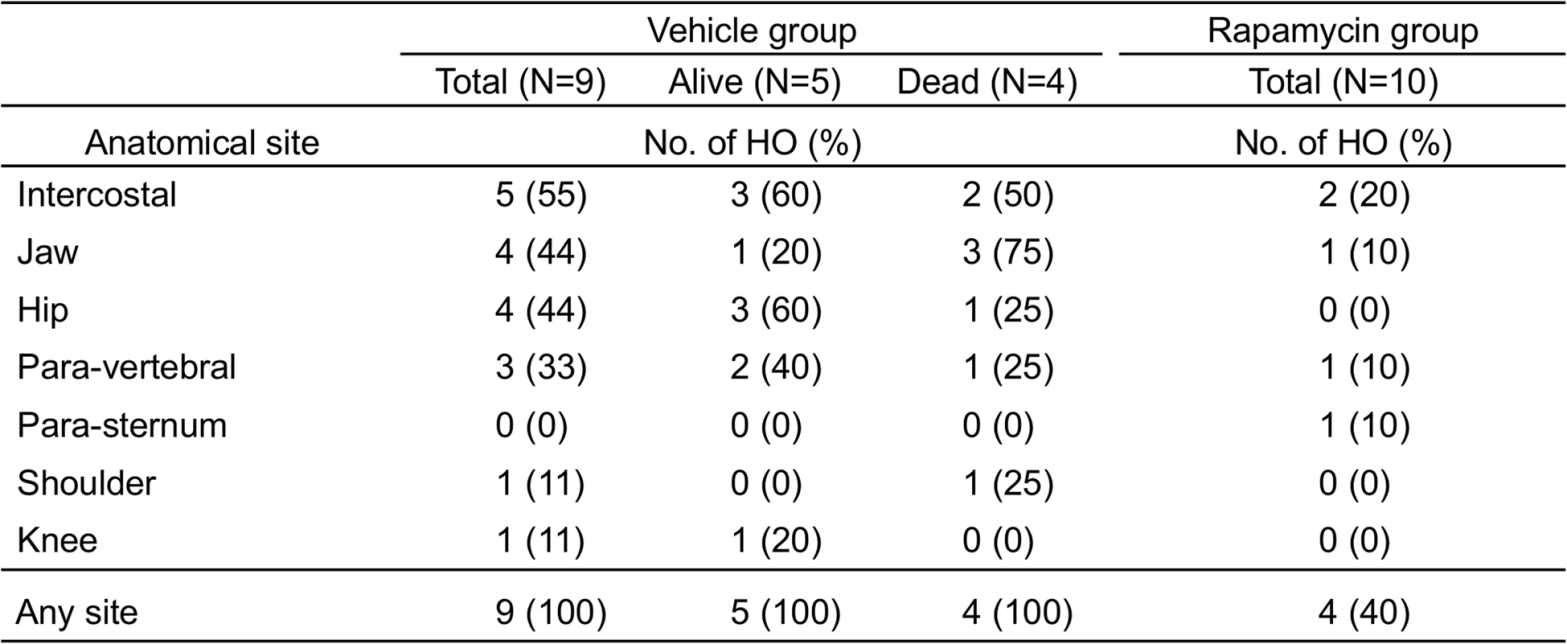
Number of HO during natural course

These results indicate that prophylactic treatment of rapamycin reduced the incidence and severity of HO during the natural course of FOP-ACVR1 mice.

### Pinch-injury induces HO in FOP-ACVR1 mice

**Fig. 2:**
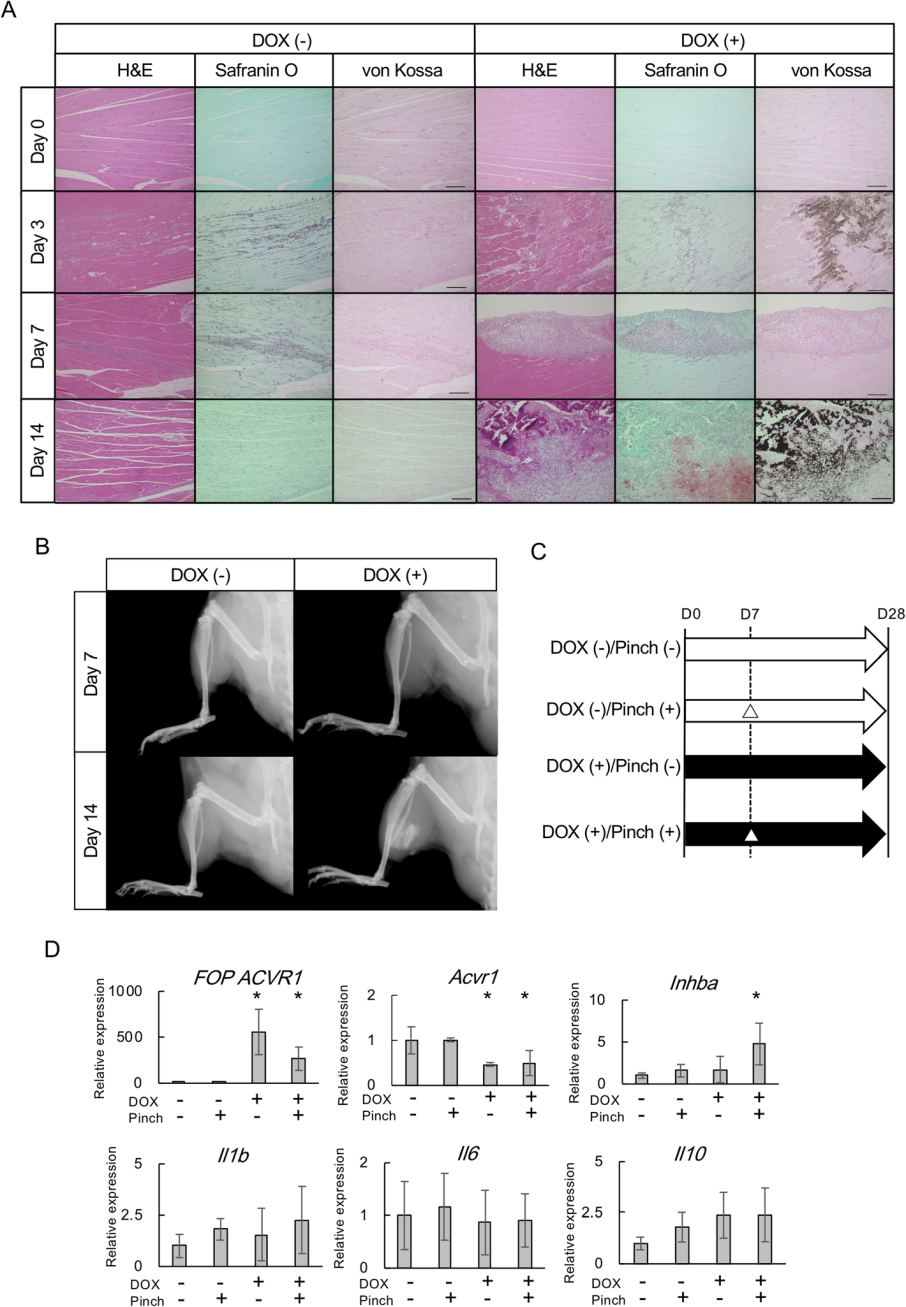
Pinch-injury induces HO in FOP-ACVR1 mice. **a** Histological findings of the injured sites. Samples were taken from the injured sites of DOX (−) or DOX (+) mice at each time point and analyzed by Hematoxylin-Eosin (H&E), Safranin O, and von Kossa staining. Scale bars = 200 μm. **b** Representative soft X-ray images of the injured limbs of DOX (−) or DOX (+) mice at each time point. **c** Schematic view of the experiments. △ indicates the day of the pinch-injury. **d** mRNA expression levels of cytokines in spleen cells at Day 28 in **c**. The expression levels in each group are relative to those of the DOX (−)/Pinch (−) group. *n* = 3 for all groups except the DOX (+)/Pinch (+) group (*n* = 4). *, *p* < 0.05, Student’s t-test

For these experiments, we divided the FOP-ACVR1 mice into a DOX (+) group, in which DOX administration was started 14 days before the pinch-injury, and DOX (−) group, in which a vehicle was administrated. Mice were sacrificed at 3, 7, and 14 days after the injury, X-ray photos of the lower limbs were taken, and muscle samples were obtained from the injured sites at each time point (Fig. 2a and b).

The histology of the skeletal muscle before the pinch-injury did not show a clear difference between the DOX (+) and DOX (−) groups (Fig. 2a, Day 0). At Day 3, DOX (+) mice showed extensive calcification in the damaged muscle cells, which was not observed in DOX (−) mice (Fig. 2a, Day 3). At day 7, although HO formation was not detected by X-ray imaging (Fig. 2b), an accumulation of fibroblastic cells and cartilage formation was observed in DOX (+) mice, but not in DOX (−) mice (Fig. 2a, Day 7). At day 14, DOX (+) mice exhibited visible bone tissues by X-ray imaging (Fig. 2b), and histological examination showed the features of endochondral ossification, which was not observed in DOX (−) mice (Fig. 2a, Day 14).

These results are compatible with previous data of HO in FOP patients [3], indicating that the pinch-injury model can be used as a model for trauma-induced HO.

### Pinch-injury induces prolonged activation of Activin-a in FOP-ACVR1 mice

To investigate the systemic effect after the pinch-injury and HO in FOP-ACVR1 mice, particularly for inflammatory cells that are known to produce Activin-A [12], we analyzed the mRNA expression of several factors in spleen cells 21 days after the pinch-injury (Fig. 2c). Mice were divided into 4 groups: DOX (−)/Pinch (−), DOX (−)/Pinch (+), DOX (+)/Pinch (−), and DOX (+)/Pinch (+). DOX (+) mice were fed with DOX from 7 days before the injury. DOX administration effectively induced the expression of *FOP-ACVR1A* in spleen cells and interestingly inhibited endogenous *ACVR1A* gene expression (Fig. 2d). It is noteworthy that the level of *Inhba* encoding inhibin beta A, which is a subunit of Activin A, was significantly higher in DOX (+)/Pinch (+) mice than in the other groups even 21 days after the injury (Fig. 2d). The expression levels of *IL1β*, *IL6*, and *IL10* were not significantly different between the four groups (Fig. 2d). To investigate the systemic change of inflammatory cytokines, the serum level of six cytokines along with Activin-A was quantified during the time course after the pinch-injury (Additional file 3: Fig. S2A; Additional file 6: Supplementary methods). In agreement with the elevated level of *inhba* mRNA in spleen cells, the serum Activin-A level was gradually increased after the pinch-injury in the Dox (+) group, but not in the Dox (−) group (Additional file 3: Fig. S2B). The serum level of the examined cytokines during the time course showed no significant difference between the Dox (−) and Dox (+) groups, except for IL-6. The serum level of IL-6 was increased at Day 3 in both groups and decreased gradually in the Dox (−) group, whereas its elevated level remained until Day 35 in the Dox (+) group (Additional file 2: Fig. S2C).

### Rapamycin inhibits HO induced by pinch-injury in FOP-ACVR1 mice

**Fig. 3:**
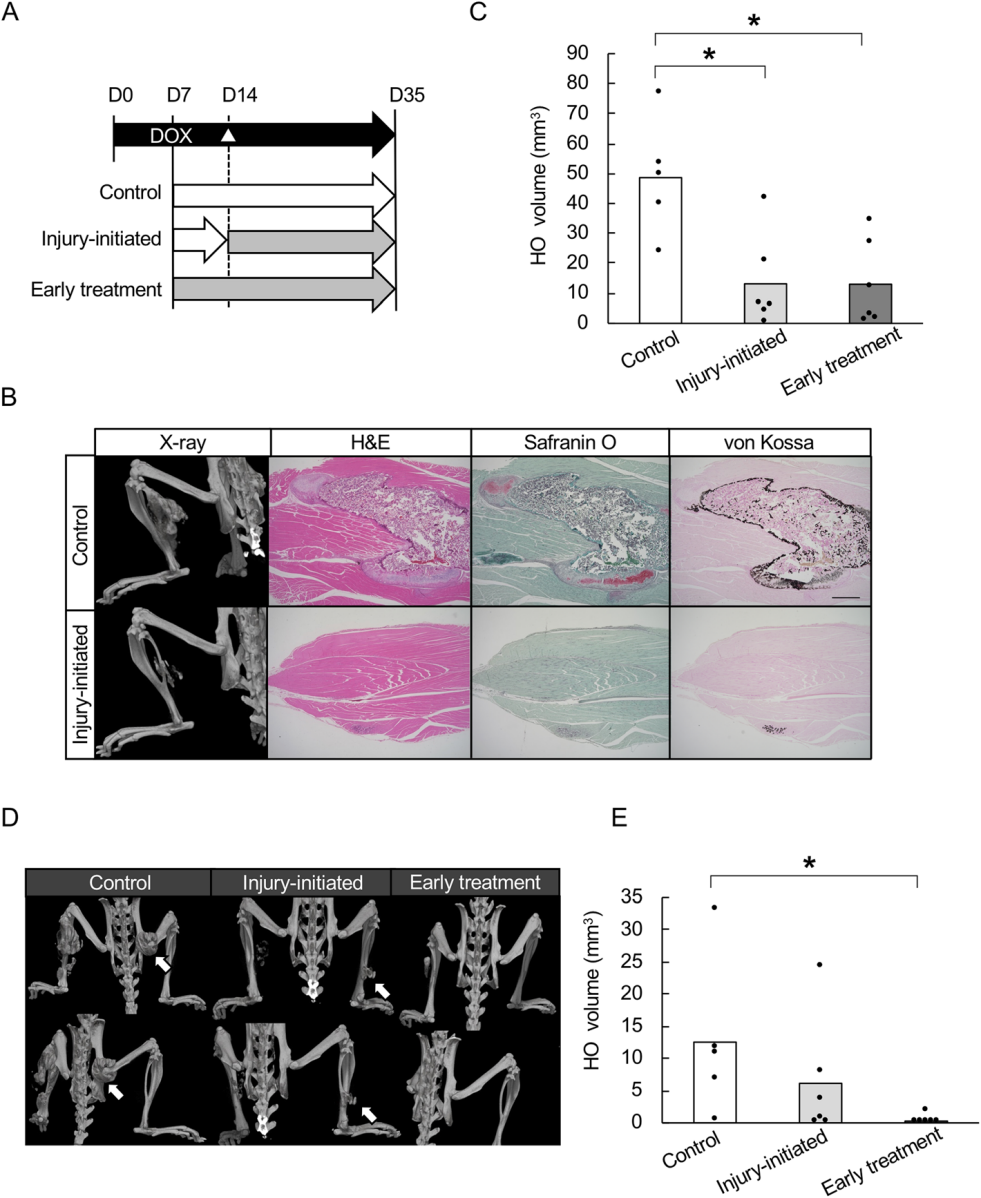
Rapamycin inhibits pinch-injury-induced HO in FOP-ACVR1 mice. **a** Schematic view of the experiments. △ indicates the day of the pinch-injury. **b** Representative μCT images and histological findings of the injured sites of mice in the control and injury-initiated groups. Tissue sections were stained with H&E, Safranin O, or von Kossa. Scale bar = 1000 μm. **c** Quantification of HO at the injured sites. The HO volume was measured from the μCT images of mice in the control (*n* = 5), injury-initiated (*n* = 6), and early treatment (*n* = 6) groups. *, *p* < 0.05, Student’s t-test. **d** Representative μCT images of HO in the contralateral limbs of mice in each group. Arrows indicate HO. **e** Quantification of HO in the contralateral limbs. The HO volume was measured from the μCT images of mice in the control (*n* = 5), injury-initiated (*n* = 6), and early treatment (*n* = 6) groups. *, *p* < 0.05, Student’s t-test

We next examined the effect of rapamycin on pinch-injury-induced HO (Fig. 3a). Seven days after the initiation of DOX administration (Day 7), FOP-ACVR1 mice were divided into three groups: 1) mice treated with vehicle until Day 35 (control group); 2) mice treated with vehicle until the day of injury (Day 14) and thereafter treated with rapamycin until Day 35 (injury-initiated group); and 3) mice treated with rapamycin until Day 35 (early treatment group).

All mice received the pinch-injury at Day 14 and were sacrificed at Day 35 (Fig. 3a). The injured sites were analyzed by μCT and histological examination (Fig. 3b). Mice in the control group developed HO at the injured-site, and histological examination demonstrated features of endochondral ossification. The size of the HO at the injured sites was much smaller in the injury-initiated group than in the control group according to μCT and histological examination (Fig. 3b). Similar findings were observed in the early treatment group (data not shown). The volume of HO in the early and injury-initiated treatment groups showed no difference, and both were significantly smaller than that of the control group (Fig. 3c).

We investigated the new HO in the contralateral limbs and found that it was formed in all mice in the control group (Fig. 3d). The incidence in the control group (100%; six out of six) was much higher than in the natural course (44%; four out of nine) after 5 weeks of DOX treatment in the same sites (hip or knee) (Table 1), suggesting an effect of the injury on HO formation at the other sites. Rapamycin treatment effectively reduced the new HO in the contralateral limbs, particularly in mice in the early treatment group (Fig. 3e).

Supposing that the inflammation at the injured site was responsible for the HO in the contralateral side, we evaluated the effect of rapamycin on the inflammatory stage of HO in FOP-ACVR1 mice. Prior to the pinch-injury, mice in the control group were treated with vehicle, and those in the early treatment group were treated with rapamycin for 7 days and sacrificed 3 days after the pinch-injury (Additional file 4: Fig. S3A). Histological examination at the injured sites revealed a massive infiltration of round cells in the control group, which were positive for F4/80, indicting monocyte/marcrophage-lineage cells. On the contrary, only few F4/80 positive cells were observed in the early treatment group (Additional file 4: Fig. S3B), indicating that the early treatment of rapamycin effectively suppressed the critical inflammatory stage of HO.

### Rapamycin inhibits the recurrence of HO after surgical resection in FOP-ACVR1 mice

**Fig. 4:**
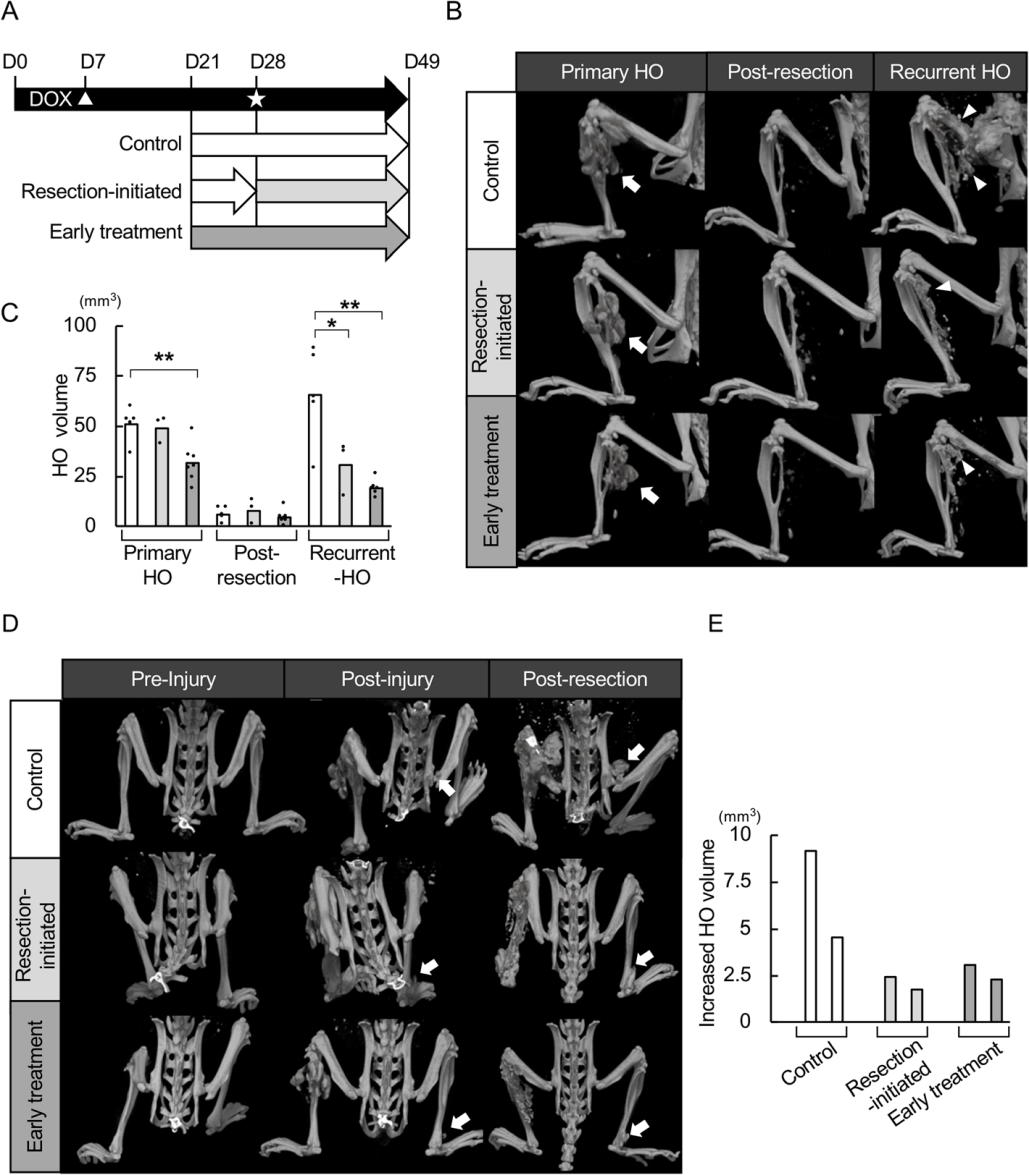
Rapamycin inhibits HO recurrence after surgical resection in FOP-ACVR1 mice. **a** Schematic view of the experiments. △ indicates the day of the pinch-injury. ☆ indicates the day of the surgical resection. **b** Representative μCT images of the injured sites in each group.. Arrows indicate injury-induced HO, and arrowheads indicate recurrent HO. **c** Quantification of HO at the injured sites. The HO volume was measured from the μCT images of mice in the control (*n* = 5), surgery-initiated (*n* = 3) and early treatment (*n* = 7) groups. *, *p* < 0.05, **, *p* < 0.01, Student’s t-test. **d** Representative μCT images of HO in the contralateral limbs of mice in each group. Arrows indicate HO. **e** Volume of increased HO in the contralateral limbs. The volume of injury-induced HO (*n* = 2 for each group) in the contralateral limbs was measured before (Day 28) and after (Day 49) the surgical resection, and the volume change was calculated

Finally, we evaluated the effect of rapamycin after the surgical removal of HO (Fig. 4a). Pinch-injury was performed 7 days after the initiation of DOX administration (Day 7), and all mice developed HO at the injured sites on Day 21 (Fig. 4b, Primary HO). Then the mice were divided into three groups: 1) mice in the control and 2) surgery-initiated groups were treated with vehicle; and 3) mice in the early treatment group were treated with rapamycin. On Day 28, the HO induced by the pinch-injury was evaluated by μCT and then resected as much as possible (Fig. 4b, Post-resection). The volume of residual HO just after the resection was recorded with μCT. In the surgery-initiated group, vehicle treatment was replaced with rapamycin treatment. Finally, all mice were sacrificed on Day 49, and recurrent HO (Fig. 4b, Recurrent HO) was evaluated with μCT (Fig. 4c) and histological examination (Additional file 5: Fig. S4). The volume of residual HO just after the resection was approximately the same among the three experimental groups (Fig. 4c). As expected at Day 49, mice in the control group showed recurrent HO with no less volume than those of primary HO (Fig. 4c). However, both the early and surgery-initiated treatment groups showed less volume of recurrent HO than the control group, with the effect being slightly better in the former group (Fig. 4c).

### Rapamycin inhibits the propagation of HO after surgical resection in FOP-ACVR1 mice

Surgical resection evokes much stronger systemic inflammation than pinch-injury and therefore may propagate systemic HO. Notably, two mice in each group showed new HO in the contralateral limbs after pinch-injury (Fig. 4d, Post-injury). The pinch-injury-induced contralateral HO became larger after the surgical resection of primary HO (Fig. 4d, Post-resection), and the increased volume of HO in control mice seemed to be larger than the volume in rapamycin-treatment mice, suggesting the rapamycin suppressed the expansion of the contralateral HO (Fig. 4e).

In summary, both early and episode-initiated treatments are effective at suppressing episode-induced HO, but early treatment seemed more effective at preventing the systemic propagation of HO, as evidenced by the observations of contralateral HO.

## Discussion

The identification of Activin-A as the initiating factor has disclosed the molecular process of HO in FOP, enabling the investigation of stage-specific targets and treatments. The first step of HO is the production of Activin-A by locally infiltrating inflammatory cells such as monocytes, macrophages, and mast cells, which can produce Activin-A by exogeneous stimuli and were reported to be abundant in the inflammatory phase of HO [12]. The genetic or chemical depletion of macrophages and mast cells in a FOP mouse model significantly suppressed HO [13], suggesting these cells as therapeutic targets. The next step of HO in FOP is the binding of Activin-A to mutant ACVR1A/ALK2 on HO-precursor cells. The binding of Activin-A to mutant ACVR1A/ALK2 expressed on the cell surface of the precursor cells can be blocked by neutralizing antibodies against Activin-A. The effect of anti-Activin A antibodies for preventing HO in natural course and also after injury was previously demonstrated in two different FOP-ACVR1 conditional knock-in mice [11, 14], and a humanized anti-Activin-A neutralizing antibody (REGN2477) is currently in a phase 2 clinical trial. The transduction of the Activin-A-induced signal can be suppressed by kinase inhibitors of the catalytic domain of ACVR1A/ALK2 such as Dorsomorphin, LDN-193189, and LDN-212854, which consequently suppresses the phosphorylation of SMAD1/5/8 proteins [15–17]. Palovarotene, an RAR-γ agonist, inhibits BMP signaling by accelerating the degeneration of SMAD1/5/8 proteins and blocks HO formation in FOP model mice expressing either Q207D or R206H mutant ACVR1A/ALK2 [18, 19]. Palovarotene is currently in a phase 3 clinical trial. Because Activin-A signals from ACVR1A/ALK2 and ACVR1C/ALK7 may activate a large number of genes, including those contributing to HO, it is difficult to pinpoint a target molecule simply by the level of expression. An alternative approach is a functional screening using a molecule responsible for the transition of HO as a surrogate marker. We identified rapamycin as a molecule to inhibit the transition of precursor cells to chondrocytes [20], which has led to a phase 2 clinical trial for FOP. Furthermore, using a later event as the screening platform, we isolated another molecule, TAK165 (mubritinib; an inhibitor of HER2 and ErbB2), as a possible therapeutic drug that indirectly inhibits mTOR activity [21].

As more drugs come to the stage of clinical trial, each drug may have a suitable time-window for exerting its optimal effect. The inhibitory effect of rapamycin on HO was initially shown to act through the inhibition of HIF-1-α, which is one of the downstream targets of mTOR [22]. HIF-1-α was identified as an upregulated molecule in patients with severe burn injury and later revealed as a key molecule for the induction of mesenchymal condensation at the injured sites, which is an initial step of HO [22, 23]. We independently found rapamycin for HO could inhibit the Activin-A-induced chondrogenesis of FOP-iMSCs [20]. In addition, we found in the present study that rapamycin inhibited the accumulation of inflammatory cells that secrete Activin-A at the injured site. These data suggest that the early phase of HO is a suitable widow for rapamycin treatment. To consider the clinical application of rapamycin, here we performed a series of experiments to investigate its effect in several clinical situations. First, we found that the continuous, episode-independent administration of rapamycin effectively inhibited HO in the natural course of our FOP-mice. We also investigated the effect for injury-initiated HO. Three days after the injury, an extensive degeneration of muscle cells with massive calcification was observed in DOX (+) mice. A similar finding was reported in extremely early lesions of human FOP [24]. At Day 7, the calcified muscle cells in our mice were replaced with chondroid tissues. This time-course may explain the result that early and injury-induced treatments showed similar preventive effects on HO, because the effect of rapamycin is expected to inhibit the transformation of precursor cells to chondrocytes [20]. Another interesting finding from the current study is that the pinch-injury induced HO in contralateral limbs in some cases (Table 1 and Fig. 3e). This event has also been reported in FOP patients and another animal model, suggesting that local injury can induce systemic signals for the HO induction in FOP [16, 25]. Although the precise mechanism of this systemic effect is not yet known, the production of Activin-A by inflammatory cells is a key event [12, 13]. The presence of inflammation in peripheral tissues can be detected in the spleen [26], and we found that the production of Activin-A and the serum Activin-A level remained high even 21 days after the injury, suggesting a vicious cycle of Activin-A and HO in FOP: Activin-A induces HO, and HO induces the expression of Activin-A in systemic inflammatory cells, which may propagate systemic HO. To prevent the HO caused by this systemic effect, early treatment seemed more effective than episode-initiated treatment. The early treatment also showed better results for the reduction of recurrent HO after surgical resection. Overall, our current data suggested that a continuous, episode-independent use of rapamycin is an effective treatment at preventing naturally developing and episode-induced HO.

It should be considered, however, that the continuous administration of rapamycin will exert adverse effects. As an immunosuppressant after kidney transplantation, rapamycin has been used for growing children for long periods, and some clinical studies reported that the long-term use of rapamycin reduced their growth velocity and induced a smaller height [27, 28]. Experiments using young rats revealed that rapamycin disrupted the proper columnar structure of the growth plate by inhibiting angiogenesis [29, 30]. Because chondrocytes in the growth plate share the process of endochondral ossification with HO, this adverse effect will be inevitable and should be seriously considered for younger patients.

It should be also noticed that the inhibitory effect of early treatment for the recurrence after the surgical resection was limited and may be insufficient in the clinical situation. Surgical resection may damage muscle tissues more gravely with massive bleeding than the pinch-injury, and thus may provoke much stronger inflammatory signals and recruit a larger number of precursor cells at the resected sites. The administration of a temporally high amount of rapamycin with or without other anti-inflammatory reagents should therefore be considered for future application.

## Conclusions

The experimental treatments demonstrated in this study, which were designed based on the clinical situations of FOP patients, will contribute to the exploration of effective treatment methods using rapamycin and also other candidate drugs for improving the quality of life of FOP patients.

## Supplementary information

**Additional file 1:Table S1.** Information of primers used in qRT-PCR

**Additional file 2:Figure S1.** Total bone volume and body weight of mice in each group

**Additional file 3:Figure S2.** Serum level of cytokines after pinch-injury

**Additional file 4:Figure S3.** Representative histological findings of the inflammatory stage of HO.

**Additional file 5:Figure S4.** Representative histological findings of recurrent HO

**Additional file 6.** Supplementary methods

## Data Availability

The data used during this study are available from the corresponding author upon reasonable request.

## Abbreviations

FOP: Fibrodysplasia ossificans progressiva
HO: Heterotpic ossification
iPSC: induced pluripotent stem cell
MSC: Mesenchymal stromal cell
DOX: Doxycycline
μCT: micro–computed tomography
H&E: Hematoxylin and eosin
