# Supplementary figures and images for: Prophylactic treatment of rapamycin ameliorates naturally developing and episode -induced heterotopic ossification in mice expressing human mutant ACVR1
Source: Orphanet J Rare Dis. 2020 May 24;15:122. doi: 10.1186/s13023-020-01406-8 (PMC7245788; doi:10.1186/s13023-020-01406-8)

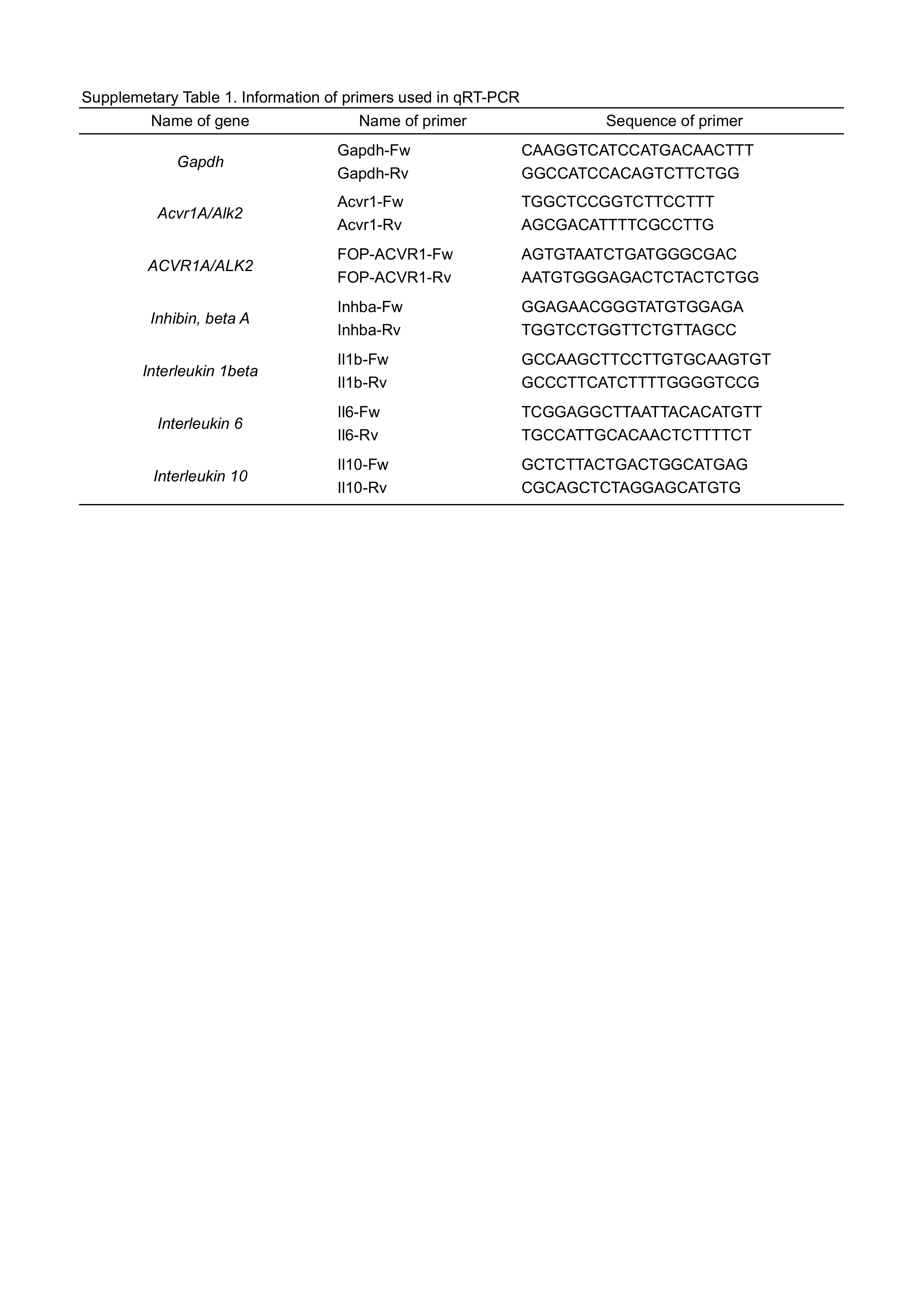

Supplement: Supplementary file 1 — Additional file 1:Table S1. Information of primers used in qRT-PCR [file 13023_2020_1406_MOESM1_ESM.tiff]

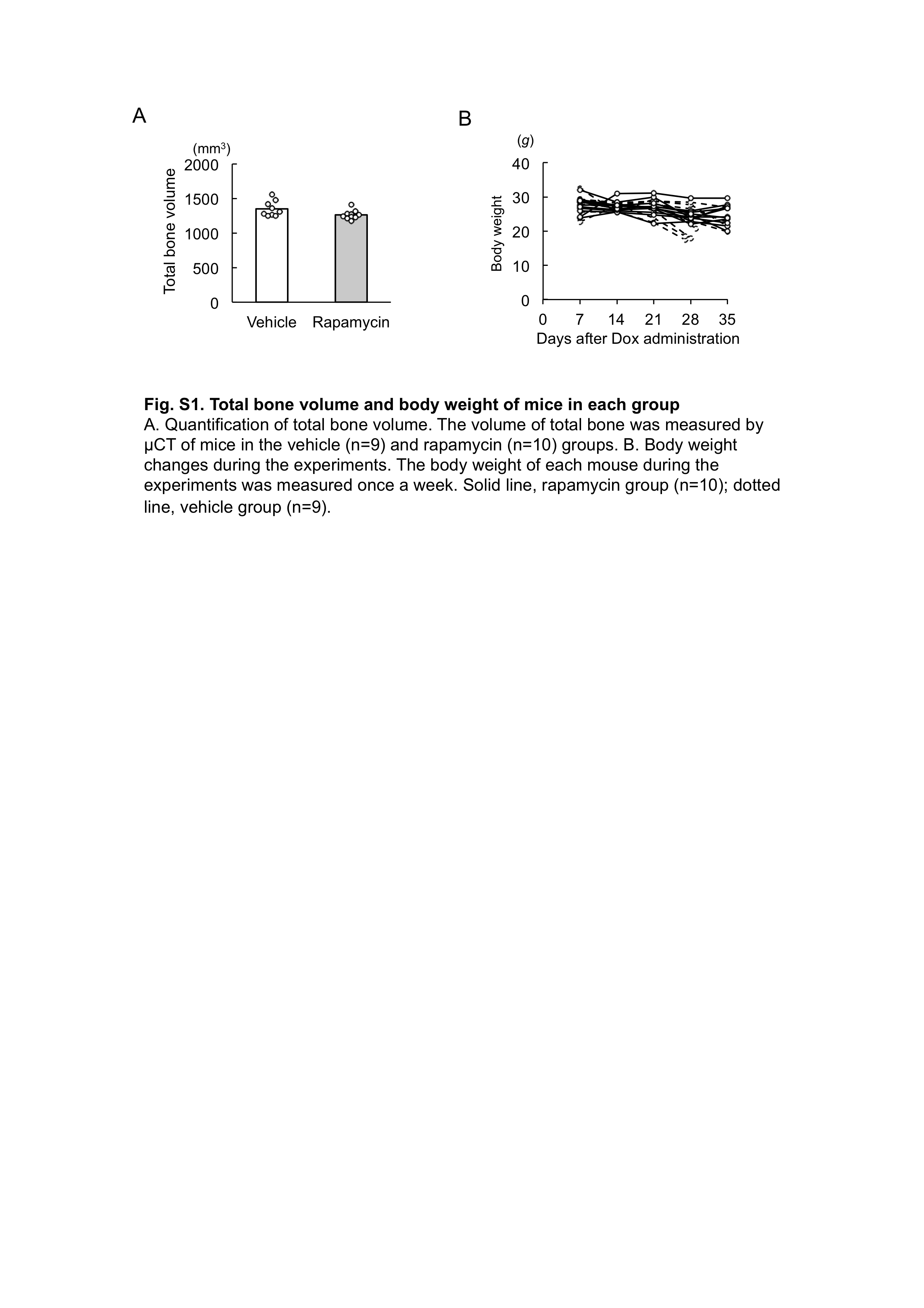

Supplement: Supplementary file 2 — Additional file 2:Figure S1. Total bone volume and body weight of mice in each group [file 13023_2020_1406_MOESM2_ESM.tiff]

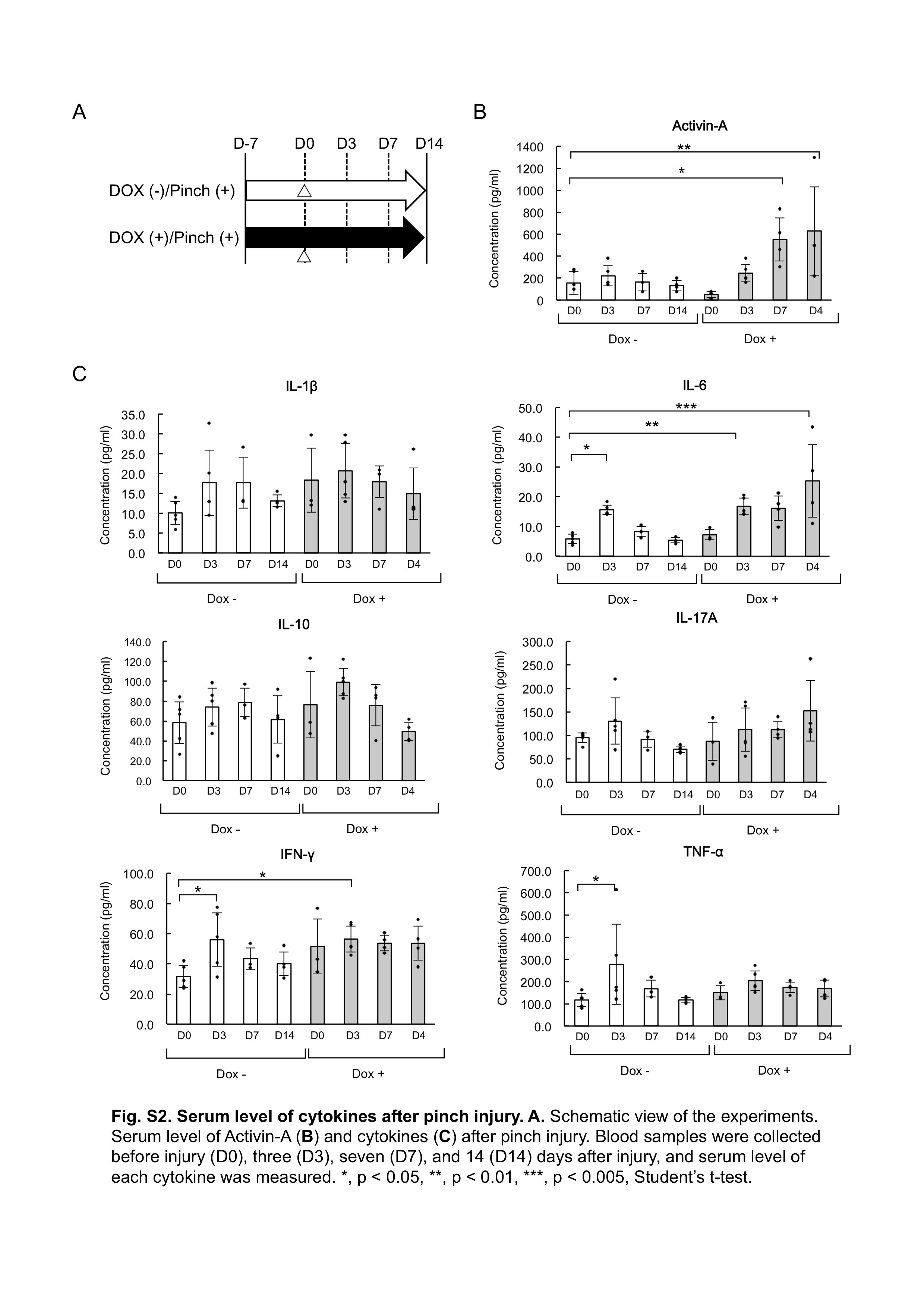

Supplement: Supplementary file 3 — Additional file 3:Figure S2. Serum level of cytokines after pinch-injury [file 13023_2020_1406_MOESM3_ESM.tiff]

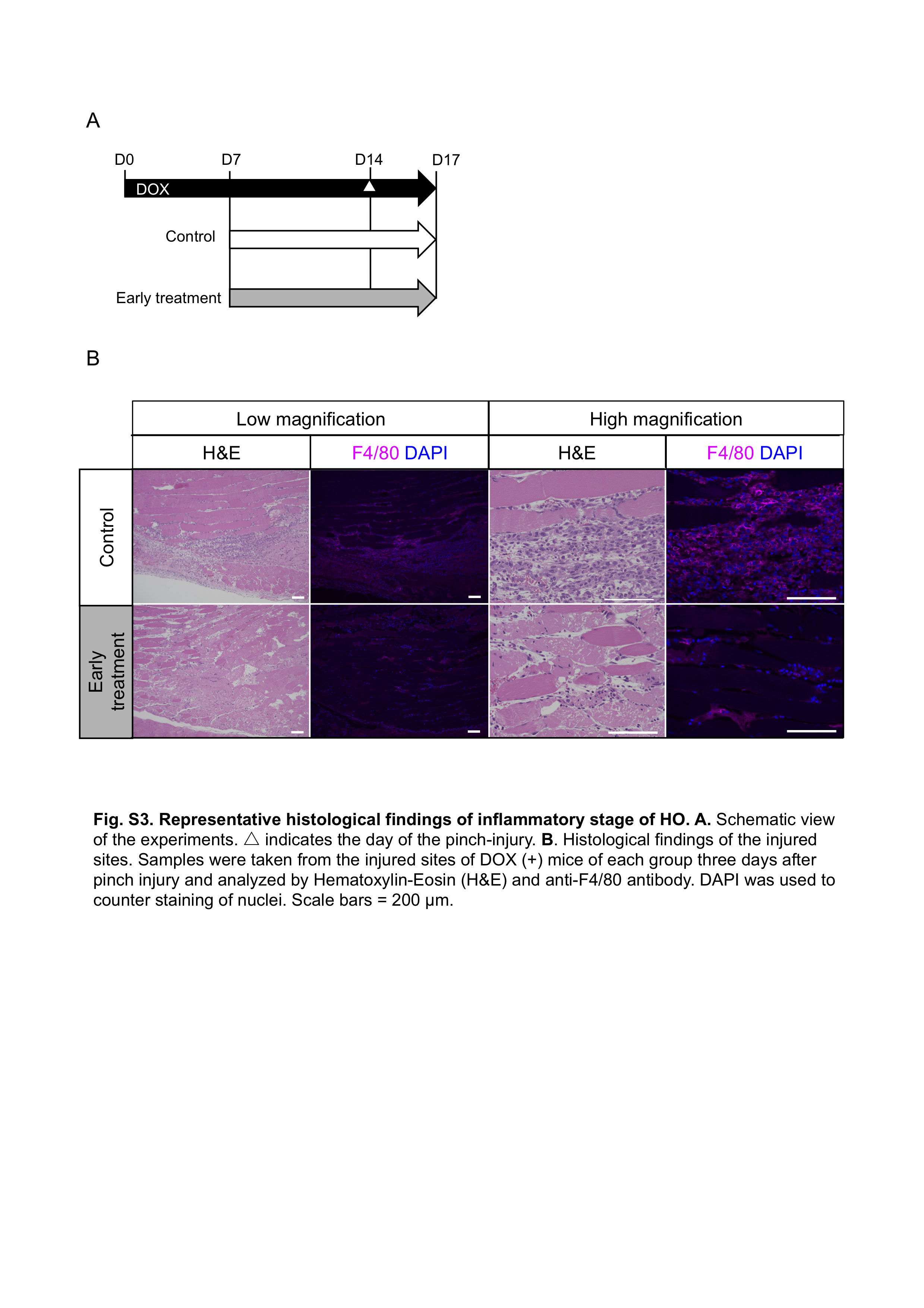

Supplement: Supplementary file 4 — Additional file 4:Figure S3. Representative histological findings of the inflammatory stage of HO. [file 13023_2020_1406_MOESM4_ESM.tiff]

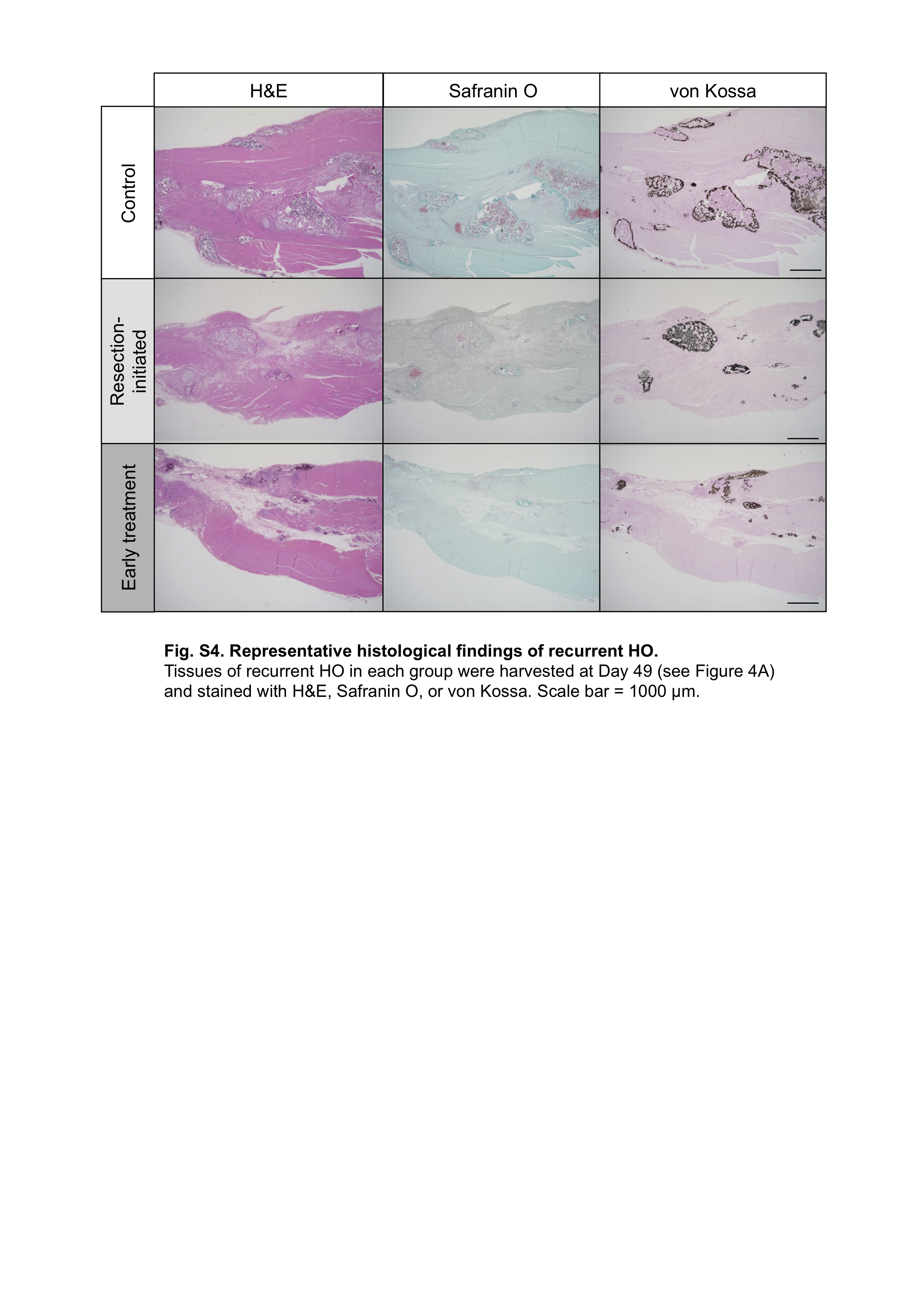

Supplement: Supplementary file 5 — Additional file 5:Figure S4. Representative histological findings of recurrent HO [file 13023_2020_1406_MOESM5_ESM.tiff]
