# Supplementary material for: Prophylactic treatment of rapamycin ameliorates naturally developing and episode -induced heterotopic ossification in mice expressing human mutant ACVR1
Source: Orphanet J Rare Dis. 2020 May 24;15:122. doi: 10.1186/s13023-020-01406-8 (PMC7245788; doi:10.1186/s13023-020-01406-8)
Supplement: Supplementary file 6 — Additional file 6. Supplementary methods [file 13023_2020_1406_MOESM6_ESM.docx]

**Supplementary methods**

*Immunohistochemical staining*

Paraffin-embedded sections were deparaffinized, rehydrated, and blocked with Blocking One (Nacalai Tesque) for 60 minutes. Slides were incubated with F4/80 antibody (Abcam; ab6640; 1:100) that was diluted in Can Get Signal Immunostain Solution B (Toyobo) for 16 to 18 hours at 4°C. Then the sections were washed three times with PBS containing 0.2% Tween-20 (Sigma-Aldrich) and incubated with chicken anti-rat IgG (H+L) secondary antibody, Alexa Fluor 647 conjugate (Thermo Fisher Scientific; 1:500) that was diluted in Can Get Signal Immunostain Solution B for 1 hour at room temperature. DAPI (10 μg/ml) was used to counterstain the nuclei. All images were obtained using a Keyence microscope (KEYENCE CORPORATION, Osaka, Japan).

*Cytokine analysis*

Blood samples were obtained from mice by cardiac puncture under deep anesthesia with isoflurane. The collected samples were kept at room temperature for 30 minutes to clot, then centrifuged at 4°C at 1000xg for 10 min to separate the serum. Serum samples were stored at -80°C until the analysis.

To quantify inflammatory cytokines in mice serum, IL-1β, IL-6, IL-10, IL-17A, IFN-γ, and TNF-α were evaluated with a Bio-Plex Pro™ Mouse Cytokine Th17 A panel (#M6000007NY, Bio-Rad Laboratories, Hercules, CA, USA). Activin-A in mice serum was assessed by the Human/Mouse/Rat Activin-A Quantikine ELISA Kit (DAC00B, R&D Systems, Minneapolis, MN, USA) following the manufacturer’s instructions and measured with an Envision multilabel plate reader (Perkin Elmer, Waltham, MA, USA).
